# Validation of the Observational Assessment Tool for Tailoring (OATT)

**DOI:** 10.1007/s11121-026-01879-2

**Published:** 2026-01-30

**Authors:** Emily S. Fu, James L. Merle, Cady Berkel, C. Hendricks Brown, Sarah Philbin, Yiqing Fan, Jenna L. McGinnis, Dania Demauro, Ariana DiGregorio, Janeth Litchey, Justin D. Smith

**Affiliations:** 1 Departments of Medicine & Psychiatry and Behavioral Neuroscience, The University of Chicago, Chicago, USA; 2 Department of Psychiatry and Behavioral Sciences, Northwestern University Feinberg School of Medicine, Chicago, IL USA; 3 Department of Population Health Sciences, Division of Health System Innovation and Research, Spencer Fox Eccles School of Medicine, University of Utah, Salt Lake City, UT USA; 4 College of Health Solutions, Arizona State University, Phoenix, AZ USA; 5 Health Sciences Integrated PhD Program, Northwestern University Feinberg School of Medicine, Chicago, IL USA; 6 Department of Psychology, American University, Washington, DC USA; 7 Department of Educational Psychology, University of Utah, Salt Lake City, UT USA

**Keywords:** Measurement development, Tailoring, Behavior change, Pediatric obesity, Implementation fidelity

## Abstract

Individually tailored interventions can address the myriad multi-level determinants of chronic health conditions. Limited measurement modalities to quantify tailoring disallow examining “active ingredient” effects on outcomes and implementation fidelity. The objective of this study is to develop and validate the Observational Assessment Tool for Tailoring (OATT) for behavioral prevention interventions. We developed the OATT and coded *n* = 172 videorecorded sessions from two trials of the Family Check-Up® 4 Health (FCU4Health), an individually tailored prevention and management program for behavioral health and obesogenic behaviors with English and Spanish-speaking participants. The sample was culturally diverse (> 65% Hispanic/Latino). Confirmatory factor analysis (CFA) tested the two-factor model. McDonald’s Omega estimated internal consistency. Discriminant and predictive validity tests were conducted with FCU4Health fidelity, engagement, and health behavior outcomes, informed by the Implementation Cascade Model. CFA confirmed a two-factor structure for both trials (i.e., RMSEA ≤ 0.06, CFI and TLI of ≥ 0.95, SRMR < 0.08 chi-square *p* ≥ 0.05). Reliability and inter-rater reliability were good (ICC > 0.77) for both trials and English and Spanish videos. The OATT was not correlated (*p* > 0.05) with discriminant validity variables. Path analysis for predictive validity indicated that fidelity to the *Individualized Treatment Planning* factor directly predicts improvements in participant engagement (*B* = 0.16, *p* = 0.01, 95% CI [0.03–0.29]), which directly predicts improvements in parent health behaviors 12 months post-baseline (*B* = 0.18, *p* = 0.01, 95% CI [0.02–0.34]). The development of the OATT is a critical step to measure and guide tailored intervention development, implementation, and evaluation. Future studies are needed to replicate predictive validity findings and test the OATT factor structure with larger samples and different prevention initiatives.

**Supplementary Information:**

The online version contains supplementary material available at 10.1007/s11121-026-01879-2.

The chronic care model and the social-ecological model explain that a person’s health outcomes are shaped by individual (e.g., psychological and behavioral), interpersonal, familial, and contextual factors (Cygan et al., 2018). Using prevention initiatives to address multi-level targets can lead to improved health outcomes. Heterogeneity in social-ecological determinants, or factors that contribute to poor health outcomes, is reflected in numerous public health issues. For example, pediatric obesity is a health-disparate public health epidemic, the most common pediatric chronic disease (Fryar et al., 2020). Intersecting multi-level determinants create challenges for prevention and management efforts, due to many moving intervention targets throughout the lifespan (Hampl et al., 2023). In a sample of *n* = 240 participants in a selective preventive intervention focused on whole child, whole family health (Smith et al., 2021), all children had body mass index (BMI) > 85th percentile at baseline but exhibited distinct profiles of potential treatment targets that included eating behaviors, physical activity levels, parenting skills, parent health behaviors, and food insecurity (Fu et al., 2020). Thus, certain families in this sample may benefit most from learning skills to increase physical activity levels, whereas others may require a greater focus on parenting skills or contextual resources to support their children’s health. Similar heterogeneity appears in the literature for physical activity itself, which is a prevention target for a multiplicity of chronic diseases such as cardiovascular disease, diabetes, and cancer. Physical activity researchers hypothesize that personalizing interventions to participants’ needs, preferences, and life circumstances demonstrate stronger effects on sustained engagement and behavior change and prevent chronic disease associated with low physical activity levels (Javed et al., 2023; Kim et al., 2025). A prevalent issue with some shared underlying etiology, substance use disorder, has reported variation in determinants in the population, such as the type of substance, behavior severity, psychiatric comorbidities, and cognitive functioning. In response, researchers have advocated for precision medicine approaches that tailor care to individual profiles and pathways of change (Carroll, 2021). At the same time, HIV prevention efforts have demonstrated that individually tailored treatment groups have achieved significantly greater reductions risky sexual behaviors compared to non-tailored treatment groups (Nyamathi et al., 2017). Together, these examples exhibit a trend that public health interventions should consider individual and contextual variability to improve behavioral risk factors and prevent associated chronic illness.

“Tailoring” is grounded in behavioral and communication theories, particularly the transtheoretical model (Prochaska & Velicer, 1997), precaution adoption process model (Weinstein, 1988), protection motivation theory (Norman et al., 2015), and communication-persuasion matrix (McGuire, 1980). Unlike a one-size-fits-all approach, tailored interventions can assess and address an individual’s experiences across multiple levels of the social ecological model as they relate to outcomes, such as individual behaviors, interpersonal relationships, access to community resources, and social determinants of health (Kreuter & Skinner, 2000). Individually tailored interventions are hypothesized to engage individuals by allowing participant choice and avoiding irrelevant material that would lead to disinterest (Patrick & Williams, 2012). Participants reported perceiving individually tailored interventions as more personal, feasibly integrated into the context of their own lifestyle, and more easily remembered compared to generic approaches (Brug et al., 2003; Ryan & Lauver, 2002). Physicians view engaging patients for a tailored action plan as an impactful strategy for facilitating health behavior change (Glasgow et al., 2023). Here, we define tailored behavioral interventions as “intervention content to address outcome-associated characteristics (e.g., family health behaviors, parenting skills, social determinants of health, mental health) that have been derived from an assessment of constructs, symptoms and metrics relevant to the intervention’s theory of change.”

Despite the theoretical support for tailoring, existing evidence for tailoring is inconsistent, which may be attributed to deficiencies in measuring how tailoring occurs. The literature about physical activity behavior change has expressed that the nascency of tailoring methodology presents a knowledge gap to developing robust, individualized intervention models for physical activity promotion (Ma et al., 2021). Formal reviews of tailored pediatric obesity interventions reported inconclusive findings due to weak descriptions of tailoring, and in response, experts urged researchers to specifically describe how interventions are tailored (Wilfley et al., 2018). In a scoping review of *n* = 69 tailored pediatric obesity interventions, *n* = 30 interventions were described as “tailored” without a formal definition or details regarding tailoring (Fu et al., 2025). Most formally defined tailored programming was digitally delivered and/or health education-centric. Of the *n* = 6 multi-component studies to address social-ecological factors, one study (Taylor et al., 2015) showed that participants receiving the “tailored package” had significant improvements in body mass index at all time points compared to the control group. In two studies, tailoring improved health behaviors, while three studies did not exhibit any differences between the tailored group and the control group. These inconsistent findings may reflect unmeasured variation in how tailoring was implemented and whether fidelity to tailoring was achieved. Fidelity is defined as “the degree to which an intervention was implemented as it was prescribed in the original protocol or as it was intended by the program developer” (Proctor et al., 2011). According to the Implementation Cascade Model (Berkel et al., 2011, 2018), facilitator delivery affects program engagement, and engagement is predictive of intervention outcomes. The model proposes that (1) lack of measurement specificity or (2) moderators inhibiting effects, rather than fidelity itself, are responsible for insignificant associations between fidelity and program outcomes. To date, there is no measurement tool to examine fidelity to tailoring. Thus, improved measurement specificity is needed to draw valid conclusions regarding tailoring’s efficacy as an intervention characteristic, and to perform adequate implementation of a tailored program (Perepletchikova et al., 2009).

The Family Check-Up® (FCU) (Dishion et al., 2008) and the Family Check-Up® 4 Health (FCU4Health), an adaptation to address whole child, whole family health in pediatric primary care, exemplify the issue of fidelity measurement for tailoring. The FCU is an individually tailored family-based intervention that utilizes an ecological assessment to tailor the treatment plan—both content and dose/duration—to improve parenting skills and parent–child relationship quality as mechanisms in the change of child behaviors and emotions. Each participating family receives a treatment plan based on their comprehensive assessment results, which typically centers on parenting modules (Dishion et al., 2012). FCU4Health was adapted from the FCU intervention to better fit within primary care by addressing whole child health (Smith et al., 2018). Families complete an ecological assessment about child and caregiver health behaviors, parenting skills, caregiver mental health, and social needs. Interventionists then review the assessment results with the family and collaboratively set 1–3 specific behavior change goals. Next, the interventionist provides a “menu” of resources and referrals to help achieve the goals. FCU4Health’s menu includes 1:1 parenting skills training, health education handouts, skills for improving eating behaviors (e.g., food logs and measuring cups), connection to mental health services for parents, referrals to social and community services, and phone check-ins. The family selects resources and referrals and schedules follow-up appointments with the interventionist to receive resources and/or check-in about referral follow through. Supplemental File 1 provides two case examples demonstrating the tailoring process and resultant tailored treatment plan.

Although tailoring has been a core component of the FCU and FCU4Health, there is no explicit measurement of an interventionist’s fidelity to tailoring, which precludes examining associations with intervention effects, parent engagement, and other processes. The COACH observational fidelity rating system (Dishion et al., 2010) for FCU and FCU4Health demonstrated evidence of predictive validity and reliability in multiple trials, but was not designed to specifically measure tailoring (Berkel et al., 2021; Chiapa et al., 2015; Smith et al., 2013a, 2013b). The COACH is rated on a 9-point scale of competent adherence in five areas: **C**onceptually accurate to the program; **O**bservant and responsive to client needs; **A**ctively structures the session; **C**orrective feedback is provided; **H**ope and motivation. Notably, tailoring is only informally embedded in the five COACH areas, and the COACH was not developed to measure tailoring, nor was it informed by tailoring theory. Overall, the measurement gap has disallowed testing if and how tailoring is an “active ingredient” and how it impacts prevention program outcomes.

Despite the theoretical promise of tailored behavioral interventions, a measurement gap negatively impacts the ability to audit tailoring and draw valid conclusions about the role of tailoring on the observed outcomes. As such, the present study aimed to:

1. Develop an empirically informed observational assessment tool called the “Observational Assessment Tool for Tailoring (OATT)” to measure an interventionist’s fidelity to tailoring.
2. Gather evidence for the reliability and validity of the newly developed tool using two trials of the FCU4Health, as illustrative examples.

To develop and validate the assessment tool, we followed Boateng et al. (2018)’s best practices for developing and validating scales for health, social, and behavioral research. The study and all procedures were approved by the Arizona State University and Northwestern University Institutional Review Boards.

## Methods

### Overview

This study used video recordings taken from two randomized trials of the FCU4Health. The FCU4Health is an ecological assessment-driven, individually tailored prevention intervention targeting whole child, whole family health in families with children aged 2–17 years. First, caregivers complete comprehensive, validated surveys about themselves (e.g., depression, parenting skills, and health behaviors), their household (e.g., food/housing insecurity and socioeconomic status), and their children (e.g., conduct behavior and health behaviors). After the assessment is complete, an interventionist meets with the caregiver for a “feedback session” to present the scored survey results. During the feedback session, the coordinator explains the meaning of each survey score, why it relates to children’s health, and how it compares to expert recommendations (e.g., CDC recommendations for physical activity). Lastly, the interventionist and caregiver set behavior change goals and collaboratively determine the treatment and follow-up plan, as described in Supplemental File 1.

The first FCU4Health trial, Raising Healthy Children (RHC), is a hybrid type 2 effectiveness-implementation study with family-level randomization to either FCU4Health or a services-as-usual condition (Smith et al., 2016). RHC included a selective prevention sample, based on elevated BMI as a risk factor for cardiometabolic conditions (e.g., type 2 diabetes mellitus and heart disease). Specifically, inclusion criteria were child aged 5.5–12 years, elevated body mass index (≥ 85th percentile for age and gender), and a consenting English or Spanish-speaking caregiver. Data were collected October 1, 2016–June 13, 2019. This study piloted the OATT using videos of families in the FCU4Health arm participating in their first feedback session in English or Spanish (*n* = 101), in which the coordinator discussed the baseline assessment results with the caregiver and collaboratively developed a treatment plan and behavior change goals. The first feedback session was chosen due to presentation of baseline assessment results and collaborative treatment planning.

The second FCU4Health trial, Healthy Communities for Healthy Students (HC4HS), is a hybrid type 2 effectiveness-implementation study with families randomized to either FCU4Health or services-as-usual (Berkel et al., 2020). HC4HS included a universal prevention sample among families in an impoverished community with disproportionate risk for obesity and mental health concerns. Inclusion criteria were an English or Spanish-speaking caregiver with a child aged 2–5 years. Study data were collected May 6, 2019–May 13, 2021. The HC4HS data were used to independently replicate and gather evidence of validity and reliability of the OATT using video recordings of a sample of families in the FCU4Health arm participating in the first feedback session in English or Spanish (*n* = 71). The total number of families eligible for inclusion in the analysis across the two studies is *n* = 172.

### Participants

The RHC sample (Berkel et al., 2020) included Latinx (69.3%), non-Latino White (14.9%), Black/African American (8.9%), Native American/American Indian (6.9%), multiple races/ethnicities (2.0%), and Asian (2.0%) caregivers with a mean age of 38.5 years (*SD* = 8.2) and their children. Children had a mean age of 9.6 years (*SD* = 1.9), 50.5% were female, and 34.7% of feedback 1 sessions were conducted in Spanish. The average child BMI-for-age-percentile was 96.0 (*SD* = 4.6) and BMI % above the 95th percentile was 116.7 (*SD* = 23.5). The average household income was *M* = $32,513.16 (*SD* = 21,414.94), 64.8% of participants reported household income > $20,000, 34.7% had private insurance, 33.7% had public insurance, 23.8% had no insurance, and 7.9% did not report insurance type. The HC4HS sample was culturally diverse and included Latinx (60.6%), non-Latino White (21.1%), Black/African American (12.7%), multiple races/ethnicities (8.5%), Middle Eastern or North African (2.8%), or Native Hawaiian or Other Pacific Islander (1.4) caregivers with a mean age of 34.2 years (*SD* = 7.4) and their children. Children had a mean age of 3.72 years (*SD* = 1.16) and 60.6% were female. Of the recorded feedback session 1 videos, 12.7% were conducted in Spanish. Approximately 67.6% of participants reported household income > $20,000, 26.7% had private insurance, 63.3% had public insurance, 2% had no insurance, and 7.0% did not report insurance type.

### Identification of Domains

First, we conducted an extensive literature review and consultation with experts in the field for identification of domains and item generation. This step focused on conceptual underpinnings of tailoring and tailored interventions, and health behavior change interventions. The experts were the developers of FCU4Health, tailored intervention researchers, and a biostatistician. Next, the preliminary two domains of the OATT were developed and defined:

1. *Assess*. The purpose of *Assess* is to measure how thoroughly an interventionist identified and understood behaviors, interpersonal relationships, and social determinants of health (Braveman & Gottlieb, 2014) that can be addressed by the intervention. Tailored treatment requires an initial assessment to understand current behaviors (Gagliardi, 2011; Ricci et al., 2022). Proper adherence to the *Assess* domain will result in an understanding of the family’s unique strengths, areas in need of support, and social determinants of health as they relate to the target outcome.
2. *Individualized Treatment Planning*. This domain’s purpose is to measure how well the interventionist facilitates collaborative treatment planning with the participant. This includes collaboration to choose areas of focus for behavior change, goal-setting (Bailey, 2019; Pearson, 2012), matching intervention resources to goals, and allowing participant choice (Charles et al., 1997; Clark et al., 2008). These mechanisms have been associated with increased intrinsic motivation, program engagement, and behavior change (Janevic et al., 2003; Patrick & Williams, 2012).

### Item Generation

Next, the items for each preliminary domain were generated through exploratory research methodologies including literature reviews and expert feedback to specify the dimensions of each domain and behaviors to score for each item.

*Assess1* is defined as follows: clarify how well the results of the comprehensive assessment reflect the participant’s experiences. The purpose of this item is to measure how thoroughly the interventionist asked the participants if their score for each assessment category (e.g., parent health behaviors, child eating behaviors, and parenting skills) is accurate and inquired about inaccuracies between the score and their lived experiences.

*Assess2* is defined as follows: point out areas of weakness for health behavior scores and allow the participant to provide more details about the areas of weakness. The purpose of *Assess2* is to measure the percentage of areas of weakness, per the assessment score, that the interventionist acknowledged and discussed with the participant to promote awareness and gather information about the behavior to inform treatment tailoring.

*Assess3* is defined as follows: point out behavioral strengths according to the assessment and allow the participant to provide more details about the strengths. The purpose of *Assess3* was to help the participant gain an understanding of their strengths and how to use them to facilitate behavior change.

*Assess4* is defined as follows: ask or allow the participantr to contextualize assessment results about mental health and social determinants of health (Braveman & Gottlieb, 2014). The purpose of *Assess4* is to measure how well the interventionist reviews the results of the comprehensive assessment’s validated mental health questionnaires (E.g., Beck Depression Inventory (Beck & Steer, 1987)) and social needs surveys (e.g., food insecurity and housing insecurity) and relates them to health behaviors. For example, the FCU4Health provides resources to meet mental health and social needs, such as counseling referrals, and assistance applying for government benefit programs. Unmet social needs and distal contextual factors affect health behaviors and introduce individual-level stressors that can also affect the interplay between individual and interpersonal interactions measured in *Assess2* and *Assess3* (Bohnert et al., 2020).

*Treat1* rates how well the interventionist guides the participant to choose areas to initiate behavior change, such as food choices, physical activity, screen time, parenting skills, or managing depression. *Treat1* does not include the actual goal-setting process, which is addressed in *Treat2*.

*Treat2* rates how well the interventionist helps the participant develop specific goal(s) based on their priorities and the intervention’s offerings (Bailey, 2019). Scores are based on how well the interventionist facilitated specific, quantifiable, relevant change goal(s).

*Treat3* measures how thoroughly the interventionist presents the different resources and referrals (e.g., parenting classes, nutrition handouts, counseling, and information about community resources) and allows the participant a choice of options. The purpose of *Treat3* is to examine how well the interventionist allows the participant to make informed choices, matched with their interest and areas of need (Deci & Ryan, 2012).

*Treat4* measures how well the interventionist informs and guides participants when determining frequency of follow-up visits, such as follow-up options, rationale for different follow-up frequencies, and allowed collaboration. It has been shown that intensive, rigid treatment schedules are often not feasible for participants, thus negatively impacting program attendance and attrition (Hampl et al., 2023). Tailored interventions could allow participants more flexibility by collaboratively determining their treatment schedule.

### Scoring Development

A codebook was drafted to identify and rate specific behaviors indicative of the degree to which interventionists enacted fidelity to tailoring (Bowling, 2014; Forgatch et al., 2005; Litwin & Fink, 1995; Priest et al., 1995; Rattray & Jones, 2007). Each item was scored on a scale of 0–5 using criterion that quantifies the presence or absence of process skills for tailoring and barriers to practice. A score of 0 on the OATT indicated the item was not done at all (adherence). If the item’s components were present, a score range of 1–5 was applied to rate the coordinator’s level of competent execution. Higher numbers reflected better fidelity and interventionist competence. A score of 3 out of 5 served as an anchor score, indicating competent completion of process skills with occasional missed opportunities. A score of 1 indicated absence of process skills and presence of barriers to practice, and a score of 5 indicated perfect execution of the item. The eight items, definitions and purpose of each item, and detailed scoring criteria for each item are shown in Table 1.

**Table 1:** OATT item descriptions and scoring criteria

Item	Behavior/process skills observed Purpose Barriers to effective practice	Scoring Criteria
		**Score (0–5**) 0 – Not done 1 – Engaged in barrier(s) to effective practice 2—Missing key process skills 3 – Adequate, the provider has an acceptable level of skill and conceptual understanding accompanied by occasional errors or missed opportunities 4 – Interventionist displays all process skills and did not miss any opportunities 5 – Excellent, mastery of process skills, above and beyond
Assess 1	How well did the interventionist ask the participant if the assessment scores accurately reflect their behaviors and if they agree with the assessment score? **Purpose:** Ask the participant how accurate the score feels compared to actual behaviors **Process skills:** • Check with participant if the score reflects their behaviors • “This is your score, does that feel accurate?” • “You responded in the survey that you do XYZ behaviors. What do you think?” • “These are your current behaviors—tell me more.” **Barriers:** • Assuming results are true without allowing participant to confirm	0 – Not done 1 – Asked < 25% of domains, engaged in barriers or 1 barrier multiple times (in a way that affected ability to assess needs) 2 – Asked ≥ 25% of domains; adequate + engaged in a barrier 3 – Adequate, the provider has an acceptable level of skill and conceptual understanding accompanied by occasional errors or missed opportunities; asked ≥ 50% of domains 4 – Asked ≥ 75% of domains 5 – Clarified how all health behavior and parenting domains from assessment reflect participant’s perceptions ☐ 0 ☐ 1 ☐ 2 ☐ 3 ☐ 4 ☐ 5
Assess 2	Point out areas of weakness according to the assessment and allow participant to elaborate **Purpose:** Gather information about areas needing attention to inform Treatment Planning **Process skills:** • Point out weaknesses (e.g., “XYZ fell in yellow zone”) • Ask guiding questions, allow elaboration • Discuss barriers, past change attempts, knowledge **Barriers:** • Cutting off, criticizing participant	0 – Not done 1 – Engaged in > 1 barrier or < 50% weaknesses pointed out 2 – ≥ 50% pointed out but no elaboration; adequate + barrier 3 – Adequate; > 50% pointed out, ≤ 50% elaborated 4 – > 75% acknowledged and > 50% elaborated on 5 – Thorough acknowledgment and elaboration on all ☐ 0 ☐ 1 ☐ 2 ☐ 3 ☐ 4 ☐ 5
Assess 3	Point out strengths according to the assessment and allow participant to elaborate **Purpose:** Help participant understand where they’re doing well **Process skills:** • Point out strengths • Allow participant to elaborate **Barriers:** • Cutting off, criticizing participant	0 – Not done 1 – < 50% pointed out; engaged in more than 1 barriers 2 – ≥ 50% pointed out, not elaborated; adequate + barrier 3 – ≥ 50% pointed out, ≤ 50% elaborated 4 – > 50% pointed out + > 50% elaborated 5 – Pointed out and elaborated on 100% of participant’s strengths ☐ 0 ☐ 1 ☐ 2 ☐ 3 ☐ 4 ☐ 5
Assess 4	Ask or allow participant to contextualize assessment results regarding mental health (MH) & Social Determinants of Health (SDOH) **Purpose:** Assess mental health and SDOH to inform planning **Process skills:** • Discuss finances, stress, mental health, housing, food insecurity • Ask if results reflect experience **Barriers:** • Cutting off, judgmental tone, exclusive yes/no questions	0 – Not done 1 – Asked < 25% MH/SDOH; engaged in barriers 2 – Asked ≥ 25% MH/SDOH; adequate + barrier 3 – Adequate, asked about ≥ 50% MH/SDOH 4 – ≥ 75% MH/SDOH + elaboration 5 – Full discussion of all MH/SDOH and participant context ☐ 0 ☐ 1 ☐ 2 ☐ 3 ☐ 4 ☐ 5
Treatment Planning 1	Prompt participant to share topics to prioritize in intervention (not specific goals) **Purpose:** Allow participant to choose topic/behavior to work on (e.g., eating behaviors, parenting skills, physical activity) **Process skills:** • Summarize assessment • Ask participant for priorities (e.g., “what do you want to work on?”) • Allow discussion **Barriers:** • Cutting off, telling them what to do, dismissive tone	0 – Not done 1 – Engaged in > 1 barrier or 1 barrier repeatedly 2 – Asked participant’s perspective but did not allow conversation about treatment preferences; adequate + barrier 3 – Adequate; allowed participant to share goals without additional prompting (e.g., “based on what we’ve talked about, what would you like your goals to be?”; gives participant sheet to write goals on their own) 4 – Allowed participant to share priorities and preferences based on assessment + back-and-forth discussion, specifically chose area(s) of priority, allowed participant to share why they chose the area 5 – Allowed participant to share priorities and their preferences with back-and-forth discussion and specific examples of intervention options ☐ 0 ☐ 1 ☐ 2 ☐ 3 ☐ 4 ☐ 5
Treatment Planning 2	Develop specific goals and/or treatment plan **Purpose:** Develop specific goals or change behaviors based on participant’s priorities **Process skills:** • Ask or allow the participant to express their preferences and thoughts to create specific goals • Allow participant to develop change goal(s) for area(s) of interest (such as area(s) selected in D1) • Ask about barriers, feasibility, what successful engagement would look like • Specific = measurable (e.g., 2x/week. Even better would be deciding which days the 2x/week will take place) **Barriers:** • Interventionist developed and set goal for participant without their input or feedback • Interventionist told participant what to do without participant input	0 – Not done 1 – Missing key process skills, engaged in barriers to effective practice. Goals are not related to what participant identifies as priority; interventionist determines goals without family input or is not responsive to family input 2 – Goals somewhat align with family priorities (e.g.,1 goal may align with priority according to participant but other(s) do not.); all goals are broad and not specific (i.e., cannot be measured); adequate + engaged in a barrier 3 – Adequate, acceptable level of skill with occasional errors or missed opportunities, e.g., created specific goals for > 1 (but not all) of participant’s priorities, did not engage in discussion about feasibility and successful engagement for all 4 – Created specific goals for all priorities according to participant preferences, discussed feasibility and successful engagement for some but not all goals 5 – Excellent, mastery of process skills, no barriers created specific goals according to participant preferences, discussed barriers, feasibility or successful engagement for all goals ☐ 0 ☐ 1 ☐ 2 ☐ 3 ☐ 4 ☐ 5
Treatment Planning 3	Interventionist gives multiple options and choice for services that are based on assessment and participant’s priorities **Purpose:** Allow participant to make informed choices based on their priorities **Process skills:** • Provide multiple options (≥ 2) • Allow participant to have choice • Provide rationale for options • Specific = measurable (e.g., 2 ×/week. Even better would be deciding which days the 2x/week will take place) **Barriers:** • Choose services for participant without their input • Gives resource while discussing priority areas and does not follow up during treatment planning (e.g., to many resources) • Interventionist repeatedly brings up a resource even though participant declined Options in FCU4Health: * Everyday Parenting* modules, community resources, health education handouts, food logs, portion plates	0 – Not done 1 – Missing key process skills, engaged in barriers to effective practice 2 – Interventionist provided 1 option; adequate + engaged in a barrier (e.g., omitted key options such as phone check ins) 3 – Adequate, acceptable level of skill with occasional errors or missed opportunities (e.g., provided list of options and allowed participant choice) 4 – Specific options (e.g., provided list of options and connected to family’s needs and preferences such as location, finances, etc. for most options and allowed participant choice) 5 – Excellent, mastery of process skills, provided list of options and connected to family’s needs and preferences such as location, finances, etc. for all options and allowed participant choice) ☐ 0 ☐ 1 ☐ 2 ☐ 3 ☐ 4 ☐ 5
Treatment Planning 4	Collaboratively decide with the participant how frequently they should meet **Purpose:** Allow participant to have informed choice of frequency of meeting based on goals and needs **Process skills:** • Explicit conversation about scheduling • Remind participant of study and intervention timeline • Propose time and ask participant about their perspective • Discuss frequency and dose of meetings **Barriers:** • Interventionist determines frequency of meeting without participant’s input • Session ends without deciding on the next meeting (this is NOT applicable if the participant has a barrier, such as moving or changing jobs, and does not know their schedule. In this case, the interventionist should schedule a time for a phone check in so they can schedule their next session)	0 – Not done 1 – Missing key process skills, engaged in barriers to effective practice 2 – Proposing a time in a yes/no question format without discussion; had discussion but did not decide on a specific date 3 – Adequate, acceptable level of skill with occasional errors or missed opportunities (e.g., explicit discussion about scheduling and interventionist includes participant when deciding next meeting (more than a proposed time + yes/no)) 4 – Conversation about scheduling including rationale for different frequency of meetings, interventionist includes participant when deciding next meeting (e.g., some people like weekly because xyz; meeting every other week could give you time to practice skills) 5 – Excellent, mastery of process skills, no barriers conversation about scheduling including rationale for different frequency of meetings, interventionist includes participant when deciding next meeting and tailors according to the family’s schedule and preferences, connects how dose matches with goals and needs, discussion about long-term and course of treatment ☐ 0 ☐ 1 ☐ 2 ☐ 3 ☐ 4 ☐ 5

### Coding Procedures

Each OATT coder was provided with the assessment results of each participating family, particularly to adequately score the *Assess* domain. When Smith (2016) used the COACH to rate coordinator fidelity to the FCU, the reliability of the COACH mean score was significantly higher when scorers were provided with participant assessment results.

### Refining and Piloting the OATT with the RHC Study

After the initial codebook was created, it was reviewed over several rounds by implementation scientists, a biostatistician, and two tailored behavior change intervention researchers with expertise in fidelity measurement. The review had a focus on content validity (Sireci, 1998), specifically, the relevance of the domains and items to the underlying construct of “individual tailoring,” representativeness, the clarity and conciseness of language, the overall scoring system, and if pertinent items were missing. Researchers piloted the codebook on two randomly selected videos to finalize the codebook instructions and items. The two videos were not included in the final analysis. Next, two coders used the finalized codebook to rate *n* = 16 videos until reliability was established—defined as 80% agreement on two consecutive RHC feedback sessions. The coders met weekly to discuss ratings and reach consensus on disagreements. After reliability was established, 20% of the remaining videos were double-coded for inter-rater reliability, and coders were masked to the double-coded assignments. A third researcher assigned and reviewed the double-coded reliability cases, and discrepancies were resolved via consensus. The coders met weekly to discuss codes and reduce drift. For sessions conducted in Spanish, three bilingual, Spanish-speaking coders double-coded *n* = 10 videos until reliability was established. Then, the three coders single-coded videos and 30% were double coded for inter-rater reliability. Discrepancies were discussed during weekly meetings to reduce drift. A total of *n* = 101 first feedback session videos were coded using the OATT, which achieved a sample size of a ratio of at least 10 participants per item for factor analysis (Tinsley & Tinsley, 1987).

### Validating the OATT and Establishing Reliability with the HC4HS Study

The HC4HS sample was used to establish the reliability of the OATT rating system and validation of the psychometric properties with an independent dataset and new group of coders. Three new coders were trained to use the OATT to score *n* = 71 videorecorded first feedback sessions in English from the HC4HS trial. The three coders first observed the RHC coders rate two videos. Next, all three HC4HS coders co-coded eight videos and discussed each video at length. Once reliability was established—defined as 80% agreement on two consecutive HC4HS feedback sessions—the coders each single-coded the remaining first feedback sessions from HC4HS with 30% double-coded to calculate reliability. The coders were all masked to the double-coded assignments. A fourth researcher reviewed the double-coded reliability cases with coders during weekly meetings to reduce drift. This process was repeated to train three bilingual Spanish-speaking coders to code the sessions (*n* = 11) conducted in Spanish.

### Measures

#### Demographics

Caregivers self-reported household income, their own, and their children’s insurance status, age, race/ethnicity, and gender.

#### Coordinator Fidelity to the FCU4Health

In the RHC trial, the COACH observational coding system measured FCU4Health coordinators’ fidelity during the first feedback session in five areas: **C**onceptually accurate to program; **O**bservant and responsive to client needs; **A**ctively structures the session; **C**orrective feedback is provided; **H**ope and motivation. Each dimension is rated on a 9-point scale of competent adherence to the program: 1–3 (needs work); 4–6 (competent work); and 7–9 (excellent work). Interrater agreement has been good in the RHC trial (≥ 0.73).

#### FCU4Health Engagement (% Recommended Services Attended)

Due to the nature of the individually tailored intervention, families received different quantities of referrals and resources. Thus, program engagement was calculated as the percentage of services recommended to families that they followed-up with throughout the intervention. Services include attending *Everyday Parenting* (Dishion et al., 2011) modules, phone check ins, and community referrals (e.g., food drives and free exercise classes).

#### Child Physical Activity (12-Month Follow-up)

In the 5-item child physical activity subscale of the Family Health Behaviours Scale (FHBS) (Moreno et al., 2011), caregivers rated the frequency of activities such as “the child participates in sports” and “plays outside” on a 5-point scale (0 = almost never, 4 = almost always). The factor model fit the data well using confirmatory factor analysis (CFI = 0.94, SRMR = 0.04).

#### Child Food and Beverage Choices (12-Month Follow-up)

Caregivers completed a 6-item scale adapted from the National Health and Nutrition Examination Survey’s Dietary Screener Questionnaire (Thompson et al., 2017) about the frequency of child food and beverage choices during the past week on an 8-point scale (0 = never, 4 = 5–6 times per week, 8 =  ≥ 6 times per day). Higher scores indicated better food choices. The factor model fit well (CFI = 1.00, SRMR < 0.05).

#### Family Mealtime Routines (12-Month Follow-up)

On the 5-item family mealtime routines subscale on the FHBS, caregivers indicated the frequency of behaviors such as “My child eats meals at the table” and “My child eats meals at a routine time.” Higher scores indicated better mealtime routines. The factor model had good fit (CFI = 0.93, SRMR < 0.06).

#### Parent Health Behaviors (12-Month Follow-up)

Parents reported frequency of behaviors such as “I keep unhealthy food out of sight,” “I work out, exercise, or participate in physical activity,” and “I serve fresh fruits and vegetables to the child” on the 10-item parent health behaviors subscale on the FHBS (CFI = 0.94, SRMR < 0.06).

### Analysis Plan

Descriptive statistics were calculated for all OATT items and all validation measures. We used confirmatory factor analysis (CFA) on the RHC sample to test a two-factor model based on the hypothesized theoretically driven framework of the OATT and to verify fit for the latent factors determined by theory. Scale items were scored using an unweighted approach (Thompson, 2004). Since the OATT uses a Likert-type scale, a weighted least squares means and variance adjusted (WLSMV) method of estimation was used (Sun, 2005) (Li, 2016). To test CFA model fit, comparative fit indices (CFI), Tucker-Lewis index (TLI), RMSEA, and chi-square were reported (Sun, 2005; Thompson, 2004). A non-significant chi-square indicated a close fit and a RMSEA ≤ 0.06, CFI of ≥ 0.95 and TLI of ≥ 0.95, and SRMR < 0.08 were considered acceptable (Cole, 1987).

To validate the OATT’s initial factor structure, we tested the CFA model on the HC4HS sample to compare the two models. Independent samples *t*-tests were used to compare differences in mean OATT item scores between trials.

Interrater reliability was calculated using a one-way random effects intraclass correlation coefficient (ICC) on absolute values, and an ICC ≥ 0.70 was considered acceptable (Bartko, 1966). The internal consistency of the scales, the degree to which the items on the scale co-vary relative to their sum score, was estimated using McDonald’s Omega (*ω*) (Hayes & Coutts, 2020).

For construct validity (Cronbach & Meehl, 1955), the extent to which the OATT accurately assesses tailoring, we conducted Pearson correlation analyses (Cohen et al., 2009) to examine how the RHC OATT scores compared to the COACH fidelity scores for the first feedback sessions. The five COACH constructs were not developed to measure tailoring. Thus, non-significant or significant negative correlation coefficients with the COACH domains suggested support for discriminant validity.

Concurrent validity, the extent to which scale scores have a stronger relationship with gold-standard measures made near the time of administration, was not completed due to lack of gold-standard measures in this field (Boateng et al., 2018; Litwin & Fink, 1995). As such, analyses will rely on internal consistency (construct validity evidence) and discriminant and predictive validity.

Predictive validity analysis was informed by the Implementation Cascade Model. We combined the two FCU4Health samples and used bivariate correlations and path analysis in structural equation modeling to examine the direct effects of OATT scores on FCU4Health engagement and the indirect effect of OATT scores on Parent Health Behaviors and Child Food Choices 12 months post-baseline with engagement as a mediator. We controlled baseline Parent Health Behavior and Child Food Choices scores. The outcome variables were selected to represent two salient child and caregiver health behaviors. A Bayesian estimator was used due to its robust performance under conditions of smaller sample sizes and non-normally distributed data compared to maximum likelihood estimator (Lee & Song, 2004) (Asparouhov & Muthén, 2010; Little & Rubin, 2002). To examine model fit using Bayesian estimation in Plus, we used the posterior predictive checking (PPC) method which is determined in two ways: (a) a 95% CI with a negative lower limit indicates good model fit and (b) a posterior predictive *p*-value (PPP) of model fit, wherein PPP values approaching zero or one indicates poor fit, and values closer to.05 suggest good fit (with.05 exactly as optimal fit) (Asparouhov & Muthén, 2010; Muthén, 2010). Descriptive statistics and interrater reliability analyses were conducted in SPSS Statistics version 29.0 (IBM Corp, 2021). CFA and path analysis were conducted using Mplus Version 8.1 (Muthén & Muthén, 2018). In Mplus, pairwise deletion is used for missing categorical variables.

## Results

### OATT Psychometrics for the RHC Sample

Table 2 shows descriptive statistics of each OATT item, and Table 3 exhibits correlations between items. All *Assess* items were correlated. CFA with correlated error terms for *Treat2* and *Treat3* showed good fit statistics (RMSEA = 0.06 (90% CI 0.00–0.12), CFI = 0.98, TLI = 0.96, *χ*^2^(18) = 25.48, *p* = 0.11, SRMR = 0.05). The *Assess* factor had good reliability (McDonald’s *ω* = 0.73) and an average score of 3.92 (*SD* = 0.71), indicating competent completion of process skills and occasional missed opportunities. *Individualized Treatment Planning* had acceptable reliability (McDonald’s *ω* = 0.64) and an average score of 3.32 (*SD* = 0.77), also indicating competent completion of process skills and occasional missed opportunities. Interrater reliability was moderate (ICC = 0.73) for videos coded in English and good (ICC = 0.81) for videos coded in Spanish. The *Assess* and *Individualized Treatment Planning* domains were correlated (*r* = 0.29, *p* < 0.05).

**Table 2:** Descriptives and independent samples *t*-tests of OATT items between the RHC and HC4HS trials

	RHC (*n* = 101)			HC4HS (*n* = 71)					
Variable	*Mean*	*SD*	*Range*	*Mean*	*SD*	*Range*	*t*(171)	*p*-value	Cohen’s* d*
Assess1	4.05	0.83	1–5	3.56	0.69	1–5	4.05	< 0.01*	0.63
Assess2	4.03	0.87	1–5	3.80	0.86	1–5	1.70	0.09	0.26
Assess3	3.85	0.85	1–5	3.00	0.94	1–5	6.18	< 0.01*	0.96
Assess4	3.74	1.28	0–5	3.04	0.89	1–5	4.24	< 0.01*	0.62
Treat1	3.79	1.02	0–5	3.49	0.80	0–5	2.04	0.04*	0.32
Treat2	2.89	0.84	0–5	2.62	0.94	0–5	1.93	0.06	0.30
Treat3	3.52	1.34	0–5	2.82	0.83	0–5	4.11	< 0.01*	0.60
Treat4	3.06	1.15	0–5	2.81	0.92	0–4	1.51	0.13	0.24
*Assess* (domain)	3.92	0.71	1–4.83	3.35	0.51	1.75–4.25	6.06	< 0.01*	0.89
*Treatment Planning*	3.32	0.77	1–4.75	2.94	0.55	1.50–4.25	3.71	< 0.01*	0.55

^*^p < 0.05

**Table 3:** Correlations between OATT items

Variable	1	2	3	4	5	6	7	8
1. Assess1	–	0.57**	0.48**	0.32**	0.13	− 0.05	0.26**	0.06
2. Assess2		–	0.49**	0.40**	0.22*	0.05	0.20*	− 0.03
3. Assess3			–	0.27**	0.16	− 0.01	0.18	− 0.04
4. Assess4				–	0.07	0.09	0.18	0.06
5. Treat1					–	0.35**	0.28**	0.19
6. Treat2						–	0.15	0.30**
7. Treat3							–	0.55**
8. Treat4								–

^*^p < 0.05; ^**^p < 0.01

### OATT Psychometrics for the HC4HS Sample

OATT interrater reliability for the HC4HS sample was good for videos coded in English (ICC =.77) and for videos coded in Spanish (ICC = 0.82). An identical two-factor CFA model from the RHC sample had good fit to the data on this sample (RMSEA = 0.04 (90% CI 0.00–0.12), CFI = 0.97, TLI = 0.95, *χ*^2^(18) = 19.74, *p* = 0.35, SRMR = 0.06).) *Assess* had an average score of 3.35 (*SD* = 0.51), indicating competent adherence and occasional missed opportunities. *Individualized Treatment Planning* had an average score of 2.94 (*SD* = 0.55), suggesting competent adherence with occasional missed opportunities and barriers to practice (Table 2). The *Assess* and *Individualized Treatment Planning* domains were correlated (*r* = 0.27, *p* < 0.05).

## Validity Test Results

### Discriminant Validity Evidence

Intercorrelations between the OATT and the COACH domains are presented in Table 4. All five COACH domains were intercorrelated. Neither of the two OATT domains significantly correlated with the five COACH domains, suggesting evidence for discriminant validity.

**Table 4:** Intercorrelations between the OATT and COACH domains in the RHC trial

Variable	1	2	3	4	5	6	7
1. *Assess*	–	0.29*	0.03	0.11	− 0.00	− 0.03	− 0.03
2. *Treatment Planning*		–	− 0.02	− 0.01	− 0.08	− 0.03	0.06
3. **C**onceptually accurate to the program			–	0.75**	0.83**	0.75**	0.30**
4. **O**bservant and responsive to client needs				–	0.78**	0.70**	0.40**
5. **A**ctively structures the session					–	0.72**	0.39**
6. **C**orrective feedback is provided						–	0.35**
7. **H**ope and motivation							–

^*^p < 0.05, ^**^p < 0.01

After combining the two samples, intercorrelations between the two OATT domains were moderate in magnitude (*r* = 0.28, *p* < 0.01), and both domain scores were used in the path analysis. *Assess* was correlated with Child Food Choices 12 months post-baseline (*r* = 0.20, *p* = 0.04) (Table 5). *Individualized Treatment Planning* correlated with Child Food Choices 12 months post-baseline (*r* = 0.19, *p* = 0.04). FCU4Health engagement correlated with Parent Health Behaviors at 12 months post-baseline (*r* = 0.20, *p* = 0.04).

**Table 5:** Intercorrelations between the OATT and health outcomes 12 months post-baseline in the combined RHC and HC4HS sample

Variable	1	2	3	4	5	6	7	8
1. *Assess*	–	0.28**	− 0.12	− 0.10	0.05	0.20*	− 0.06	− 0.03
2. *Treatment Planning*		–	0.11	0.12	0.03	0.19*	− 0.12	0.05
3. FCU4Health Engagement			–	0.20*	0.11	− 0.10	0.15	0.13
4. Parent Health Behaviors				–	0.47**	0.20*	− 0.07	0.45**
5. Family Mealtime Routines					–	0.10	− 0.02	0.35**
6. Child Food Choices						–	− 0.12	− 0.02
7. Child Beverage Choices							–	0.04
8. Child Physical Activity								–
*M*	3.68	3.16	0.79	2.95	3.43	4.29	2.05	2.72
*SD*	0.69	0.71	0.34	0.50	0.56	1.38	1.18	0.76
*Range*	1.00–5.00	1.00–4.75	0.00–1.00	1.90–4.00	1.60–4.00	0.67–7.67	0.00–5.67	0.40–4.00
*Valid N*	172	167	172	125	125	125	124	125

^*^p < 0.05, ^**^p < 0.01

**Fig. 1:**
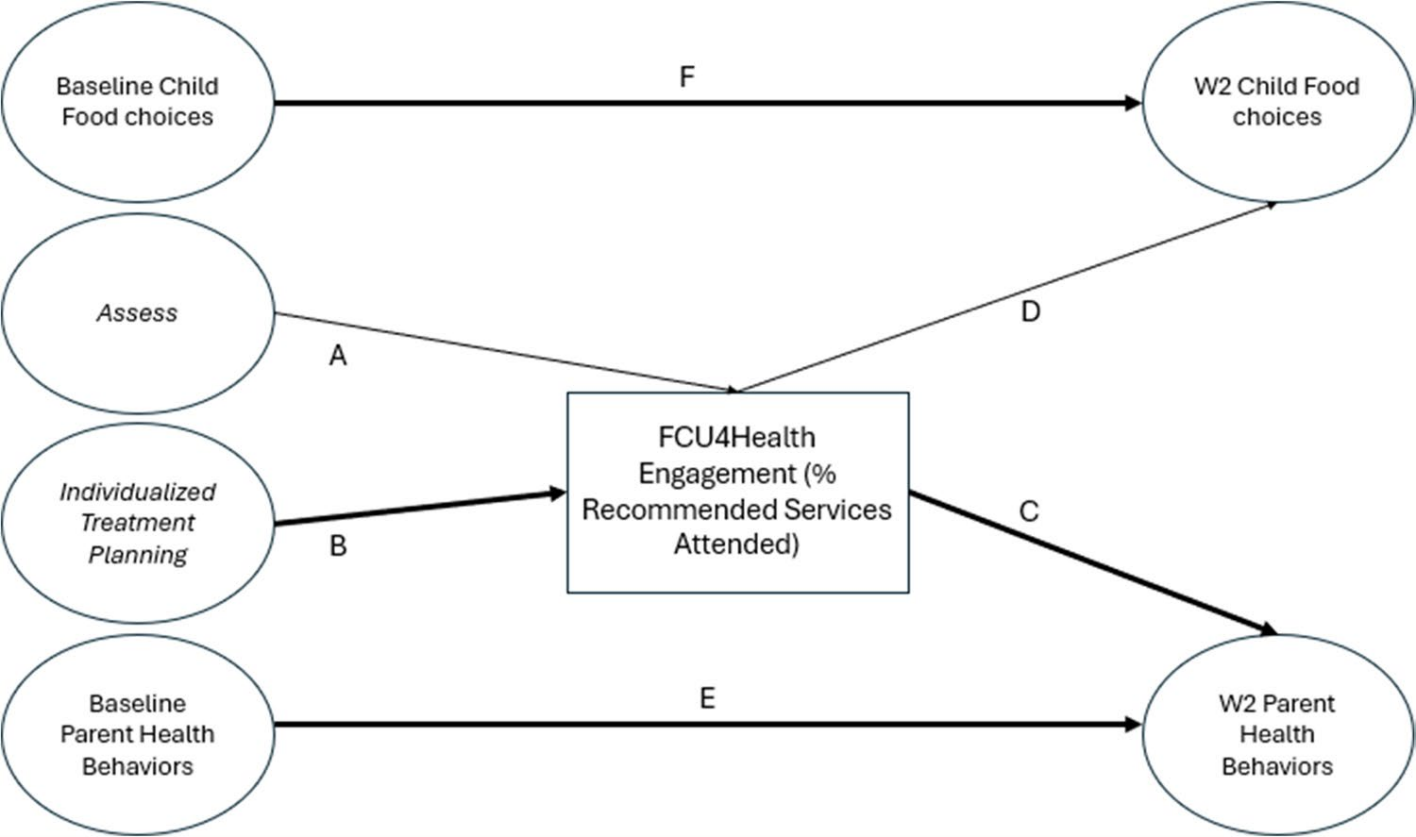
Conceptual model: The relationship between OATT domains, FCU4Health engagement, Parent Health Behaviors and Child Food Choices 12 months post-baseline. Paths in bold are significant. W2, 12 months post-baseline; *Assess*, *Assess* domain; *Treat*, *Individualized Treatment Planning* domain

The path analysis model, based on the Implementation Cascade Model, had good fit to the data: PPC (95% CI [− 16.00–20.48]; PPP (0.43). The path coefficients for the model in Fig. 1 are provided in Table 6. The *Individualized Treatment Planning* domain predicted FCU4Health engagement (*B* = 0.16, *p* = 0.01, 95% CI [0.03–0.29]), and engagement predicted Parent Health Behaviors 12 months post-baseline (*B* = 0.18, *p* = 0.01, 95% CI [0.02–0.34]). The indirect path between *Individualized Treatment Planning* and Parent Health Behaviors, mediated by engagement, was significant (*B* = 0.02, *p* = 0.02) (Table 7). The indirect path between *Assess* and Parent Health Behaviors, mediated by engagement, neared significance (*B* =  − 0.03, *p* = 0.05, 95% CI [− 0.08–0.01]), in the opposite direction.

**Table 6:** Results of path analysis of the two outcome model

Model path	*B*	Posterior SD	*p*-value	95% credibility interval
A. *Assess* → Engagement	− 0.16	0.09	0.03	− 0.34, 0.02
B. *Treat* → Engagement	0.16	0.07	0.01	0.03, 0.29
C. Engagement → W2 Parent Health Behaviors	0.18	0.09	0.01	0.02, 0.34
D. Engagement → W2 Child Food Choices	−0.05	0.08	0.24	− 0.22, 0.08
E. Baseline Parent Health Behaviors → W2 Parent Health Behaviors	0.49	0.06	≤ 0.001	0.34, 0.60
F. Baseline Child Food Choices → W2 Child Food Choices	0.56	0.05	≤0.001	0.34, 0.65
***R***^**2**^				
Engagement	0.04	0.03	≤ 0.001	0.01, 0.13
W2 Child Food Choices	0.32	0.06	≤ 0.001	0.21, 0.42
W2 Parent Health Behaviors	0.28	0.07	≤ 0.001	0.14, 0.40

W2, 12 months post-baseline; *Assess*, *Assess* domain; *Treat*, *Individualized Treatment Planning* domain; Engagement, participant % recommended services attended

**Table 7:** Indirect effects between the OATT domains, engagement, and parent health behaviors 12 months post-baseline

Indirect effects	*B*	Posterior *SD*	*p*-value	95% credibility interval
*Assess* → Engagement → W2 Parent Health Behaviors	− 0.03	0.02	0.05	− 0.08, − 0.01
*Treat* → Engagement → W2 Parent Health Behaviors	0.02	0.02	0.02	0.01, 0.09

W2, 12 months post-baseline; *Assess*, *Assess* domain; *Treat*, *Individualized Treatment Planning* domain; Engagement, participant % recommended services attended

## Discussion

This study aimed to develop and assess the reliability and validity of an observational assessment tool measuring interventionist fidelity to tailoring, the OATT. Factor analysis confirmed a two-factor structure for the OATT and McDonald’s *ω* indicated adequate reliability. Correlation and regression models showed evidence of discriminant validity and partial evidence of predictive validity according to the Implementation Cascade Model (Berkel et al., 2011, 2018). Intra-class correlations revealed evidence of good interrater reliability in both English and Spanish, accounting for the challenges of training coders to use a video observation assessment tool for a multicomponent intervention. Thus, initial analyses of the OATT suggest that it may be a promising tool to measure interventionists’ fidelity to tailoring in prevention interventions.

The RHC and HC4HS trials had significant mean differences between their OATT scores, which were consistently lower in the HC4HS sample compared to the RHC sample. One explanation could be the trial timing, as the RHC trial recruitment and follow-up sessions were completed by the year 2018, whereas HC4HS was in the middle of recruitment and feedback sessions when the COVID19 pandemic was declared in March 2020. This disruption likely presented challenges when reviewing the social-ecological assessment results, reflected in the lower HC4HS *Assess* item scores. Another consideration is the different targeted populations and associated procedural differences between the two trials. RHC tested FCU4Health with families of children with elevated BMI (e.g., selective prevention). HC4HS tested FCU4Health as universal prevention, recruiting families of children ages 2–5 who did not have a BMI-based inclusion or exclusion criterion. Procedurally, all families enrolled in HC4HS were offered parenting sessions regardless of assessment results, as exposure to parenting modules when children are younger has demonstrated efficacy as a prevention strategy for children’s later health outcomes (Smith et al., 2015). As such, this may explain lower scores for all four *Individualized Treatment Planning* items and the OATT captured such procedural differences.

To evaluate predictive validity, we used path analysis in structural equation modeling to examine the role of tailoring according to the Implementation Cascade Model. Results demonstrated partial support for this model in that one dimension of the OATT, *Individualized Treatment Planning*, predicted higher program engagement (% recommended services attended), which predicted improved parent health behaviors at 12 months post-baseline. Specifically, selecting areas to prioritize, informing the participant of the intervention offerings, and matching intervention resources to participant’s areas of interest during the first feedback session increased the likelihood of engaging in the follow-up programming throughout intervention enrollment. These items were developed based on self-determination theory, such that facilitating autonomy, competence, and relatedness during an intervention can facilitate motivation and behavior change (Deci & Ryan, 2012; Patrick & Williams, 2012). On the other hand, the *Assess* domain had a near significant relationship with engagement, in the opposite direction as hypothesized. It is possible that thoroughly reviewing the assessment took time away from self-determination theory aligned behaviors, which decreased participant motivation to follow through with resource referrals. Additionally, an intersection between the two domains could be at play, as assessment of results informs the collaborative treatment planning. Future studies should examine if a cut-point representing competent demonstration of the *Assess* domain is sufficient.

The OATT has potential to address existing challenges of measuring fidelity in multicomponent, multiple outcome-tailored interventions. Although the OATT was only evaluated using two trials of the FCU4Health, it aspires to be adapted for different health outcomes that have psychosocial and behavioral determinants. For example, treatment for public health issues such as substance use, physical activity promotion, and HIV prevention have called for tailored approaches in response to the heterogeneity of determinants (Nyamathi et al., 2017). The OATT provides an explicit definition of tailoring as “intervention content to address outcome-associated characteristics (e.g., family health behaviors, parenting skills, social determinants of health, mental health) that have been derived from an assessment of constructs, symptoms and metrics relevant to the intervention’s theory of change.” The OATT aims to promote uniform adherence to tailoring and competency of core components (Brown et al., 2015; St. George et al., 2019; Wilfley et al., 2018). Future studies may explore using this definition and adapting the OATT for other prevalent chronic illness and behavioral prevention initiatives.

### Limitations

Although the current study has important implications for tailored interventions, the results must be interpreted in light of several limitations. Although studies have shown a range of sample size requirements depending on the complexity of the CFA model (i.e., 30 to 460 cases) (Kyriazos, 2018; Wolf et al., 2013), it is accepted that small sample sizes allow problems to arise, such as inaccurate parameter estimates and model fit statistics (Wang & Wang, 2019). This may have been present in the HC4HS sample. Finally, the Spanish videos were not used to refine the codebook during the development process. Although this may have led to missed opportunities, the inter-rater reliability between Spanish coders was good in both samples. As with any newly developed measurement tool, future studies with larger sample sizes are needed to gather more evidence of validity and reliability for the OATT.

Additionally, the study’s use of secondary data was limiting because the study can only follow the parameters set by the original RHC and HC4HS studies. The researchers of the current study were potentially not aware of protocol changes that could affect measured variables during secondary analysis (Johnston, 2014). However, the secondary data were appropriate for piloting the newly developed OATT such that video recordings and survey data were entirely available, and the intervention content and design were ideal to test the OATT. Furthermore, the different FCU4Health trials allowed leveraging existing data to test and validate the observational assessment tool, a design that allows behavioral health services and implementation research to be conducted more efficiently (Chambers et al., 2010). Future research should use the OATT to design and carry out a tailored intervention study to better characterize tailoring and examine effects on process and outcome variables.

## Conclusion

This study introduces a measurement tool to guide tailored intervention development and measure interventionist fidelity to tailoring. As the OATT is a newly developed tool that is one of its kind, additional studies are needed to validate the OATT’s factor structure with larger samples and programs that target different health outcomes other than pediatric obesity. Future research is needed to further examine the hypothesis that tailoring, when done with competency, can positively affect a participant’s engagement in program offerings and health behavior change. Next iterations should work toward better establishing predictive validity, simplifying the OATT to enhance opportunities to assess fidelity to tailoring and push engaging and effective tailored behavioral prevention programs upstream.

## Supplementary Information

Below is the link to the electronic supplementary material.

ESM1 (DOCX 24.5 KB)

## Data Availability

This study was not formally registered. The analysis plan was not formally pre-registered. De-identified data from this study are not available in a public archive. De-identified data from this study will be made available (as allowable according to institutional IRB standards) by emailing the corresponding author. Analytic code used to conduct the analyses presented in this study are not available in a public archive. They may be available by emailing the corresponding author.Some of the materials used to conduct the study are presented in a public archive: https://psychology.asu.edu/reach/familycheckup4health.

## Declarations

### Clinical Trial Number

This is a secondary analysis using data from the following two clinical trial registration numbers: NCT03013309 ClinicalTrials.gov and NCT04469816 ClinicalTrials.gov.
