# Supplementary material for: Validation of the Observational Assessment Tool for Tailoring (OATT)
Source: Prev Sci. 2026 Jan 30;27(2):316–32. doi: 10.1007/s11121-026-01879-2 (PMC12999605; doi:10.1007/s11121-026-01879-2)
Supplement: Supplementary file 1 — (DOCX 24.5 KB) [file 11121_2026_1879_MOESM1_ESM.docx]

**Case Examples**

**Example 1**

**Participants:**

- Female Caregiver/Biological Mother, referred to as “Mother.”
- Biological father, lives at home with the family, referred to as “Father.”
- 11-Year-old male, referred to as “Child.”

The family was referred to FCU4Health by their pediatrician.

**Ecological assessment results:**

- The assessment results showed that the health-related areas that need attention (red) were family health routines (specific to media use and mealtime routines), child’s eating behaviors and beverage choices.
- Mother endorsed depression and anxiety in her assessment.

**Discission of results:**

During the “feedback session,” the interventionist first discussed the ecological assessment results with the Mother, Father, and Child. Mother and Father agreed with the assessment results that family media use and mealtime routines, and Child's health behaviors are of concern and would like to change them. Mother shared that the kids watch TV and play games on their tablets at night before bed. The children also tend to watch TV during dinnertime and they family does not eat together at the table. Mother and Father both had a hard time saying “no” to Child when he wants high-calorie snacks or asks for sugary beverages. Father works night shifts and feels too tired to say “no” to Child, and feels guilty about not being present. He feels it is easier to allow Child to have sugary beverages, even though he knows it is negatively affecting Child’s health. Mother has healthy habits for her own diet, but less around physical activity. She reports having a strong relationship with Child, but Child disagreed.

The interventionist also pointed out the mother’s depression and anxiety responses on the questionnaires. Mother responded that she was already connected with a mental health provider at the time of the interview, and she is enjoying therapy but anticipates that it may become difficult to attend sessions due to caring for the children.

After reviewing the ecological assessment results with Mother and Father, the interventionist summarized what they discussed, and asked Mother and Father to set 1-3 goals and a follow-up appointment according to what they consider 1) most important and 2) most achievable. Mother and Father decided that they would like to improve their family media use and mealtime routines, and the improved structure could also improve the child’s eating behaviors and beverage choices. Next, the interventionist discussed with Mother and Father reviewed their current behaviors, and what they’d like to change. The parents stated they would like to put away electronic tablets before mealtime and bedtime, and to eat dinner together at the table 2-3x/week (an increase from 0x per week). However, they expressed low confidence about how they would enact this and communicate this to Child and his siblings.

In response, the interventionist explained that learning new parenting and communication skills may help achieve their behavior change goals. She introduced the *Everyday Parenting* modules and their connection with health behavior change: *discussing positive behavior support* (being proactive and offering incentives to reward the behavior we want to see more of), *monitoring and limit setting* (to monitor and set limits between healthy and unhealthy routines), and discussing *relationship quality* (strengthening the parent-child relationship, communication skills, co-parenting, and establishing family routines that support healthier behaviors). Mother and Father agreed that parenting skills would be helpful and would like to meet every other week for a 1:1 session to learn *limit setting* skills, and other parenting skills they decided would be helpful afterward, such as *relationship quality*. At the end of each follow-up session, they will decide if they would like to schedule another follow-up appointment to continue learning the module, learn new skills, set new goals, and/or stop meeting.

The interventionist informed Mother and Father of the other FCU4Health offerings, including resource materials and handouts, such as the FCU4Health [Healthy Behavior Guidelines](https://drive.google.com/file/d/1P4viFjT_am3CaFMNSqqXNt0kUqKAmAQo/view?usp=drive_link) handout, and food logs to track intake. The family felt that their existing behavior change goals around screen time and routines were a good start, and declined the resource materials. They accepted the healthy behavior guidelines handout to reference later, but they were already aware that their behaviors fell short of health behavior guidelines and didn’t think that reviewing additional information during the session would be beneficial.

Regarding Mother’s mental health, the interventionist asked Mother if she could check in to see if Mother is still receiving mental health services, and help Mother problem-solve any barriers to engaging in treatment. The interventionist provided psychoeducation about how caregiver wellbeing can impact individual and children’s health behaviors, and how attending therapy can assist with her goals of improving their family health routines. Mother agreed to biweekly phone check ins to discuss progress on their goals, and to discuss and problem-solve barriers/facilitators to engaging in her mental health treatment.

The family did not endorse any difficulties affording physical activity or nutritious eating opportunities, and thus the interventionist did not review referrals to free community resources.

**Treatment Plan**: FCU4Health interventionist will call Mother every other week to check in about goal progress and engagement in mental health services. They will problem-solve barriers to mental health care if needed. The interventionist will meet with Mother and Father for three follow-up appointments, every other week, to teach *Everyday Parenting* modules about *limit setting*, and any additional modules they would be interested in.

**Example 2**

**Participants:**

- Female Caregiver/Biological Mother, referred to as “Mother.”
- Biological father, lives at home with the family, referred to as “Father.”
- 8-Year-old male, referred to as “Child.”

The family was referred to FCU4Health by their pediatrician.

**Social-ecological assessment results:**

- Mother and Father scored in the green zone on parenting warmth, their own health behaviors and family health routines, family stress and parent well-being
- The feedback form showed that the health-related areas that need attention (red) were child’s eating behaviors, food choices, emotional adjustment, and coping & self-management
  - Child’s beverage choices were in the green zone

**Discission of results:**

During the “feedback session,” the interventionist first discussed the results with Mother, Father, and Child. Mother shared that Child’s weight and health behaviors are very important to her and she is ready to make changes. Although they saw that Child’s physical activity, eating behaviors, and food choices were in the “red zone,” they stated that they do not know what the food and physical activity recommendations are for children. The interventionist provided the FCU4Health [Healthy Behavior Guidelines](https://drive.google.com/file/d/1P4viFjT_am3CaFMNSqqXNt0kUqKAmAQo/view?usp=drive_link) handout about expert recommendations for 8-year-old children’s physical activity and fruit/vegetable intake. Mother and Father agreed that Child is not meeting the recommendations and would benefit from improvement. The interventionist also pointed out the family’s green zones and collaboratively discussed ways they could use their strengths to model good health behaviors for Child, and use their strong relationship to facilitate eating behavior and physical activity change. Mother and Father felt unsure about how to add more fruits/vegetables and decrease intake of unhealth food. Thus, the interventionist made notes to discuss resources during treatment planning to help improve their confidence.

After reviewing the ecological assessment results with Mother and Father, the interventionist summarized what they discussed, and asked Mother and Father to formally set 1-3 goals and a follow-up appointment according to what they consider 1) most important and 2) most achievable. Mother and Father decided that they would like to improve their Child’s physical activity levels and food choices. Next, the interventionist discussed with Mother and Father reviewed their current behaviors, and what they’d like to change, using small, specific changes to increase chances of sustained change. The parents decided to increase Child’s vegetable intake to 2-3 vegetables/day. They also set a goal to take a 20 minute family walk in the morning 2-3x/week (an increase from 0x/week).

When asked, Mother and Father expressed low confidence about how they would work toward their goals, especially given Child’s difficulty with emotional adjustment and coping.

In response, the interventionist explained that learning new parenting and communication skills may help achieve their behavior change goals. She introduced the *Everyday Parenting* modules and their connection with health behavior change: *discussing positive behavior support* (being proactive and offering incentives to reward the behavior we want to see more of), *monitoring and limit setting* (to monitor and set limits between healthy and unhealthy routines), and discussing *relationship quality* (strengthening the parent-child relationship, communication skills, co-parenting, and establishing family routines that support healthier behaviors). Mother and Father agreed that learning how to do “positive behavior support” would help Child create new habits around food choices and physical activity. Mother and Father scheduled a follow-up session with the interventionist to learn *the Positive Behavior Support* module and how to use it to support Child’s health behavior changes. to build on their existing parenting skill strengths. The parents reported a busy schedule, especially with additional caregiver duties, and they agreed to meet with the interventionist monthly to learn new skills.

The interventionist informed Mother and Father of the other FCU4Health offerings, including resource materials and handouts, such as health education about screentime and sleep, food logs to track intake, and phone check-ins to assess goal progress. The family felt that their existing behavior change goals around physical activity and increasing vegetable intake were a good start, and declined the additional resource materials. They were interested in phone check-ins to increase accountability, and opted for weekly 5-10 minute phone check-ins to discuss goal progress and problem-solve barriers that arise.

The family did not endorse any difficulties affording physical activity or nutritious eating opportunities, and thus the interventionist did not review referrals to free community resources. Furthermore, the parents agreed with the assessment results that portrayed their family stress and parent well-being in the “Green zone.”

**Treatment Plan**: FCU4Health interventionist will call Mother every week to check in about goal progress. They will problem-solve barriers to mental health care if needed. The interventionist will meet with Mother and Father for monthly follow-up appointments to teach *Everyday Parenting* modules about *positive behavior support.* At the end of the follow-up appointment, they will decide if they would like to meet again to improve skills, learn new skills, or set new goals.
